# High-flow nasal cannula in the real world: powerful support, hidden severity

**DOI:** 10.62675/2965-2774.20260012

**Published:** 2026-03-16

**Authors:** Israel Silva Maia, Domenico Luca Grieco, Jean-Pierre Frat

**Affiliations:** 1 Research Institute Hospital do Coração São Paulo SP Brazil Research Institute, HCor-Hospital do Coração - São Paulo (SP), Brazil.; 2 Hospital Nereu Ramos Florianópolis SC Brazil Hospital Nereu Ramos - Florianópolis (SC), Brazil.; 3 Universidade Federal de Santa Catarina Florianópolis SC Brazil Universidade Federal de Santa Catarina- Florianópolis (SC), Brazil.; 4 Catholic University of The Sacred Heart Department of Anesthesiology and Intensive Care Medicine Rome Italy Department of Anesthesiology and Intensive Care Medicine, Catholic University of The Sacred Heart - Rome, Italy.; 5 Fondazione Universitario A. Gemelli IRCCS Department of Emergency and Intensive Care Medicine and Anesthesia Rome Italy Department of Emergency and Intensive Care Medicine and Anesthesia, Fondazione Universitario A. Gemelli IRCCS - Rome, Italy.; 6 Centre Hospitalier Universitaire de Poitiers Médicine Intensive Réanimation Poitiers France Médicine Intensive Réanimation, Centre Hospitalier Universitaire de Poitiers -Poitiers, France.

High-flow nasal cannula (HFNC) has become a cornerstone of contemporary respiratory support.^(1,2)^ Initially introduced as an easy-to-use, simple, and comfortable alternative for respiratory support in hypoxemic respiratory failure in the intensive care unit, its indications have expanded rapidly across hospital settings, patient populations, and clinical contexts.^(3-6)^ Randomized clinical trials have established HFNC as an effective therapy in selected populations.^(3,4,7)^ However, subsequent studies suggest that HFNC may not be uniformly superior to other noninvasive respiratory supports used in acute respiratory failure, with evidence indicating potential advantages of alternative strategies in specific clinical contexts – such as helmet noninvasive ventilation in hypoxemic patients and facemask noninvasive ventilation in acute hypercapnia.^(8)^ Yet, how HFNC is deployed and performs at scale in routine clinical practice outside the context of randomized trials remains poorly investigated. In this context, the large, system-wide observational study by Bouhassira et al.^(9)^ presented in this issue offers timely and important insights into the real-world use of HFNC.

Leveraging electronic health record data from more than 28,000 episodes of HFNC use across a five-hospital system over eight years, this study provides a rare and precious overview of HFNC practice.^(9)^ The most striking finding is the steady and substantial increase in HFNC use between 2017 and 2025, underscoring how rapidly this therapy has been adopted. High-flow nasal cannula is no longer a niche intervention confined to intensive care units or narrowly defined indications; rather, it has evolved into a default respiratory support strategy across diverse hospital environments.

Although the landmark trial by Frat et al.^(3)^ was published in 2015 and established HFNC as an effective therapy for acute hypoxemic respiratory failure, widespread adoption appears to have lagged behind the initial evidence. As illustrated by the temporal trends in this study, the most pronounced increase in HFNC use occurred during and after the COVID-19 pandemic, when utilization peaked and continued to rise steadily thereafter. This acceleration likely reflects a convergence of factors, including greater clinical familiarity and the emergence of additional supportive evidence across diverse contexts of respiratory failure.^(4,5)^ Together, these observations suggest that the pandemic catalyzed integrating HFNC into routine practice at scale, well beyond its initial trial-based indications.

Yet, alongside this expansion in use, the study reveals outcomes that are notably less favorable than those reported in many randomized trials. Approximately one-quarter of HFNC episodes were followed by intubation, and nearly 30% by death or hospice discharge. At first glance, these figures may appear sobering and could be misinterpreted as evidence of limited effectiveness. However, such a conclusion would overlook a central lesson of real-world data: HFNC is frequently applied in patients with high illness severity and often as an escalation therapy rather than as an early intervention.

Beyond patient-level factors, one of the most compelling contributions of this study is the demonstration of substantial hospital-level variation in outcomes. The authors report that 16% of the variation in mortality and 20% of the variation in intubation rates were attributable to differences between hospitals. Such findings challenge the notion that HFNC outcomes are determined solely by patient characteristics or disease severity. Instead, they point to the profound influence of institutional context – local protocols, monitoring bundles, and clinician experience, thresholds for escalation, staffing models, and resource availability.

This variability echoes a broader theme emerging across critical care research: interventions do not exist in isolation. Their effectiveness is inseparable from the systems in which they are deployed. High-flow nasal cannula, in particular, is highly sensitive to timing, monitoring, and decision-making around escalation. Prolonged HFNC use outside of closely monitored environments may delay necessary intubation in some patients,^(10)^ while early escalation in others may expose them to avoidable invasive ventilation. The challenge lies not in choosing HFNC *versus* intubation, but in identifying the right patient, at the right time, in the right setting.

High-flow nasal cannula is a powerful tool that likely allows clinicians to support, and sometimes "mask" the severity of respiratory failure in very sick patients, making outcomes look worse simply because the treated population is intrinsically high-risk.^(11)^ Since the evidence that brought HFNC into guidelines largely comes from trials in critically ill patients, these real-world data that included non-critically ill patients warrant caution for its routine use in clinical practice: HFNC may falsely reassure clinicians about patients’ severity and delay escalation if not paired with rigorous monitoring and clear failure criteria.

The study also brings renewed attention to treatment heterogeneity. Although HFNC is used across a wide range of acute respiratory failure phenotypes and care settings, this analysis in this study is limited by the lack of granular phenotypic classification and phenotype-specific outcomes, which are important given that clinically meaningful endpoints and escalation thresholds differ across conditions such as de novo hypoxemia, cardiogenic pulmonary edema, and post-extubation support. As a result, the study cannot clarify whether HFNC effectiveness varies across these subgroups, reinforcing the need for future studies that incorporate phenotype-specific definitions of success and failure.

Importantly, this work does not undermine the value of HFNC. Rather, it underscores the need for thoughtful clinical governance. As HFNC becomes ubiquitous, health systems must develop clearer protocols defining indications, monitoring requirements, and criteria for treatment failure. Integrating HFNC into structured escalation pathways – alongside noninvasive ventilation and invasive mechanical ventilation – may help reduce unwarranted variation and improve outcomes. Moreover, real-time decision-support and risk-stratification tools could help clinicians identify patients unlikely to benefit from prolonged HFNC therapy.

In summary, this study provides a valuable and sobering reminder that HFNC is not merely a device, but a clinical decision embedded within complex patient and institutional contexts. Its outcomes are shaped as much by where and how it is used as by its physiological effects – particularly the availability of close monitoring and the discipline to escalate promptly when response is inadequate. As HFNC continues to proliferate across hospital systems, the focus must shift from whether it works on average to when, where, and for whom it is most likely to improve patient-centered outcomes. Only with this contextualized perspective can HFNC truly deliver on its promise in modern respiratory care.
